# *In Vitro* Propagation of Muña-Muña (*Clinopodium odorum* (Griseb.) Harley)

**DOI:** 10.1155/2012/196583

**Published:** 2012-10-18

**Authors:** María Soledad Diaz, Lorena Palacio, Ana Cristina Figueroa, Marta Ester Goleniowski

**Affiliations:** CEPROCOR, Science and Technology Ministry, Arenales 230, X5004APP Córdoba, Argentina

## Abstract

A micropropagation protocol was developed which may assist in the safeguarding and augmentation of dwindling natural populations of *Clinopodium odorum* (Griseb.) Harley, a critically and endangered medicinal plant. Factors affecting culture initiation bud sprouting and growth, rooting, and acclimatization were studied, using nodal segments of *in vitro* germinated seedling as primary explants on six media supplemented with different concentrations and combinations of 6-benzylaminopurine (BAP) (0.5–1.5 and 2-Naphthalene acetic acid (NAA) (0.5–1.5). Best results for culture initiation with sustainable multiplication rates (100%) were obtained on WP medium without any growth regulator. WP with the addition of 0.5 : 1 or 0.5 : 1.5) of BAP and NAA promoted a higher elongation; however, the optimum number of nodes were obtained in plantlets grown on 1/2 MS with the addition of 1 : 1.5 of BAP and NAA. Culture of sectioned individual nodes transferred to the media with different rates of BAP and NAA 1/2 MS-9 (1.5 : 1.5), SH-8 (1.5 : 1.0), and 1/2 B5-4 (1.0 : 0.5) media resulted in no proliferated shoots. The *in vitro* plants were successfully acclimatized garden soil and sand (2 : 1) in the greenhouse, with over 90% survival rate. The *in vitro*-grown plants could be transferred to *ex vitro* conditions and the efficacy in supporting *ex vitro* growth was assessed, with a view to develope longer-term strategies for the transfer and reintroduction into natural habitats.

## 1. Introduction

*Clinopodium odorum* (Griseb.) Harley is a small deciduous shrub of the family Lamiaceae commonly known as muña-muña. In Argentine, the species is restricted to a very specific niche; plant exploration studies in the region has revealed the occurrence of only small populations that is especially characteristic of Pampa de Achala (Córdoba) distributed at an elevation of 1200 m [1].

The fresh herb is used as a flavoring agent for aliments and an infusion of the aerial parts is utilized as an anticatarrhal, antispasmodic, stringent, carminative, digestive, diuretic, laxative, stomachic, soporific, vermifuge, menstrual suppression, flatulent, colic, and tonic digestive and antispasmodic and to help in parturition [2, 3].

This plant species have been excessively collected from its habitats and become endangered due to different contributory factors: extensive denudation of the forest floor, caused by cattle grazing and collection of leaf litter, and removal from the wilderness which is highly used in the preparation of liquor companies “Amargos serranos” [4, 5].

Seed is only the means of propagating *C. odorum,* but seeds have reduced probability of germination when adult plants are already growing in the area, thus reducing species dissemination [6]. 

The use of *in vitro* techniques for rapid and mass propagation offers possibilities for recovery of endangered species thus reducing the risk of extinction. This technique could enable production of large numbers of clonal plants in relatively short-time periods using very little starting material [7]. 

The establishment of *in vitro* germplasm banks in developing countries has great importance, but these techniques must be associated with other plant genetic resources conservation practices [8, 9]. The *in vitro* conservation techniques allow material exchanges among germplasm banks, and the germplasm keeps its sanitary conditions and viability during the transport [10, 11]. 

To our knowledge, there are no reports for *in vitro* propagation of *C. odorum*; it is known that developing efficient micropropagation procedures for particular species generally requires detailed studies to define specific composition of mineral salts, plant growth regulators, and organic compounds in the culture medium.

In view of the importance of this species, a holistic approach for the propagation using *in vitro* methods for its conservation have been described in this paper.

## 2. Materials and Methods

### 2.1. Plant Material

Seeds of *C. odorum* were collected in 2010 in their natural habitat during the months of February and March when the fruits are ripened and kept at room temperature until the initiation of the experiments. A voucher specimen was deposited in the International Herbarium of the National University of Río Cuarto, Argentine.

### 2.2. Seeds Germination. 

For *in vitro* germination different conditions were assessed: (A) control, (B) washing overnight in running tap water, (C) soaking at 1 mg L^−1^ of Gibberellic acid (GA_3_) during 12 h, (D) soaking at 10 mg L^−1^ GA3 during 12 h, (E) soaking at 100 mg L^−1^ GA_3_ during 12 h, (F) washing overnight in running tap water plus soaking at 1 mg L^−1^ GA_3_ during 12 h, (G) washing overnight in running tap water plus soaking at 10 mg L^−1^ GA_3_ during 12 h and (H) washing overnight in running tap water plus soaking at 100 mg L^−1^ of GA_3_ during 12 h. 

Seeds were surface sterilized with a solution of 70% (v/v) ethanol for 2 min and rinsed 3 times with sterile distilled water, followed by 15 min in a solution of sodium hypochlorite (NaOCl) 1.5% (v/v) for 15 min and finally rinsed 3 times with sterile distilled water. These surface sterilized seeds were explanted onto Murashige and Skoog, 1962 (MS) [12], germination culture medium, supplemented with 3% (w/v) sucrose, 0.7% (w/v) agar, and pH 5.8, using 20 seeds/lasks. Culture tubes were incubated at 25 ± 2°C for 15 days in darkness. Germination rates were measured 15 days after starting the culture. The plantlets were kept under a photoperiod of 16 h of cool white fluorescent light (30 *μ*E m-2S-1) followed by 8 h darkness. 

Each treatment (in triplicate) consisted of 20 seeds and germination was monitored weekly up to 10 weeks. 

### 2.3. Culture Initiation. 

Clonal plants (plants originally derived from seed) were initially propagated on media for 4-5 months to increase stock populations for multiplication rate assessment. Multiplication experiments were carried out using nodal segments with axillaries' buds of *in vitro* germinated seedling aged on half-strength MS major and minor salts medium with 1 mg L^−1^ of indole-3-butyric acid (IBA) (initiation medium). 

The regeneration potential of nodes with axillaries' buds (0.5–1 cm) obtained from plants grown in the above *in vitro* culture condition was evaluated in terms of frequency (survival percentage), principal shoot length, nodes number, shoot formation, and rooting by culturing on six media: MS, Schenk and Hildebrandt, 1972 (SH) [13], Lloyd and McCown, 1980 (WP) [14], Gamborg et al., 1968 (B5) [15], and MS and B5 at half-strength salt medium (1*⁄*2 MS and 1*⁄*2 B5), containing 3% (w/v) of sucrose and supplemented with 6-benzylaminopurine (BAP) and 2-naphthalene acetic acid (NAA) at different concentrations (0.5–1.5 mg L^−1^) and combinations. The pH media was adjusted to 5.6 with NaOH or HCl, prior to gelling with 0.7% (w/v) agar-agar, dispensed (10 mL) into culture tubes and sterilized by autoclaving (121°C for 15 min.). In all the experiments, the chemicals used were of analytical grade (Sigma-Aldrich, St. Louis, MO, USA and E. Merck, Darmstadt, Germany). 

All cultures were maintained under culture conditions: 25 ± 2°C, 45–55% relative humidity, and 16 h photoperiod under cool white fluorescent light (30 *μ*E m-2S-1). Explants were observed daily for the first week for signs of contamination and thereafter weekly for signs of growth and development.

### 2.4. Acclimatization

Young plants with well-developed roots were carefully removed from the glass culture vessels; the roots washed with sterile water to eliminate the excess of agar and transferred to plastic cups containing autoclaved garden soil and sand (2 : 1). Each pot was covered with plastic bag for plantlets acclimatization during 15 days, which was punctured the last 5 days to allow air exchange. 

Each plantlet was irrigated with distilled water every 2 days for 2 weeks followed by tap water for two other weeks. The potted plantlets were initially maintained under culture room conditions (4 weeks) and later transferred to normal greenhouse conditions. 

### 2.5. Statistical Analysis

All experiments were conducted at least three times. For each treatment, a minimum of 14 and a maximum of 25 explants were used. For statistical analysis, all quantitative data expressed as percentages were first submitted to arcsine transformation and the means corrected for bias before a new conversion of the means and standard error back into percentages [16]. Statistical analysis was performed by ANOVA, and significantly different means were identified using Duncan's test *P* ≤ 0.05.

## 3. Results and Discussion

### 3.1. Effect of Different Strategies on Seed Germination

Many studies evaluating adequate *in vitro* conditions for propagation of several species have been conducted using *in vitro*-germinated plants, to avoid disinfection of explants, because of extreme sensitivity to common disinfection procedures and subsequent low survival rates [17, 18]. A seed was scored as “germinated” when the root emerged and grew to 1 cm. *C. odorum* seeds were capable of germinating in all the treatments used, although several rates of germination were obtained. 

As shown in Table 1, only at 28.1% germination was observed in the control. It is known that in some plant species, seed dormancy is a condition which prevents the seed from germinating, even when it is perfectly healthy and all conditions for germination are at an optimum. 

Seed dormancy is also a prevalent cause of very slow and erratic germination in the majority of wild plants [19–22].

Therefore, strategies to induce physiological and mechanical seed dormancy rupture were carried out, which caused a positive reaction on the *C. odorum* seed germination response. The washing overnight in running tap water condition was not enough mechanism to prevent total seed dormancy, with the resulting germination rate being only 41.8%. Germination rate is the “speed or velocity” of germination and can be expressed as the time it takes for a defined percentage of seed to germinate in our case at 15 days.

More than 2-fold improvement in germination was recorded in seeds washing overnight in running tap water plus soaking at 1 mg L^−1^ GA_3_ during 12 h, in which a physiological dormancy release increasing germination rates at 62.2% was obtained (Table 1).

This germination value was similar to those obtained with soaking 1 mg L^−1^ GA_3_; it suggests that *C. odorum* seeds had among others a requirement to GAs which is known that induce the production of enzymes to digest the endosperm that would otherwise from a mechanical barrier to radicle emergence reported that the use of this plant growth regulator (GA_3_) is an improved alternative to release dormancy seed; this induction mechanism resulted to be more effective on other species [19, 22, 23].

### 3.2. Plant Tissue Culture

Successful plant tissue culture depends on the choice of nutrient medium. The explants of most plant species can be grown on completely defined media and there is a small number of standard culture media that are widely used with inorganic supplements. No single medium can be used for all types of plants and organs, so the composition of the culture medium for each plant material has to be worked out [24]. Best results for culture initiation with sustainable multiplication rates (100%) were obtained on WP medium without any growth regulator.

Significant differences were observed among the media for shoot elongation (Table 2). The maximum shoot length was significantly higher on hormone-free media WP and SH media resulting 7.74 ± 0.68 and 7.46 ± 1.14 cm, respectively (Table 3). 

A scarce elongation (2.37 ± 1.44 cm) was observed when the explants were placed on 1*⁄*2 B5. The plants produced on 1*⁄*2 MS showed significant differences for the number of nodes per plant among other treatments resulting with a value of 9.46 ± 0.91 at *P* ≤ 0.05.

The media 1*⁄*2 MS-9, SH-8, and 1*⁄*2 B5-4 were not effective to induce growth and caused necrosis, thus resulting in death of nodal segments.

The steps in plant tissue culture protocols can impose a series of PGRs; in many plants, a balance between two groups of PGRs; auxins and cytokinins, constitutes the most conspicuous group of plant hormones regulating cell division and elongation and determining morphogenesis. An adequate assessment of the suitability requirement of PGRs depends upon the type of plant tissues or explants that are used for culture. 

 The use of culture media supplemented with BAP and NAA has been associated with *in vitro* morphogenetic events and it is known that cytokinins have the ability to induce the shoot formation.

Mc Cown and Sellmer, 1987 [25], reported that the effect of growth regulators can be strongly modified by the medium on which the culture is grown. When the experiments were conducted to determine how different ratios of plant growth regulators would support microplant growth, significant difference with an excellent growth response (length of principal shoot and node number) corresponded to plantlets grown on 1*⁄*2 MS-6 (12.14 ± 0.64 cm) and WP-2  (12.67 ± 0.66 cm) and WP-3 (12.61 ± 0.74 cm). 1*⁄*2 B5-3 affected significantly shoot proliferation as it recorded the highest number of new shoots/explants, 7.0 ± 0.58 after 8 weeks in culture (Table 3). 

A rooting rate of 90% was attained when the plantlets were placed in inductive media or included in hormone-free, suggesting NAA incorporated to the media was not necessary. 

**Figure 1:**
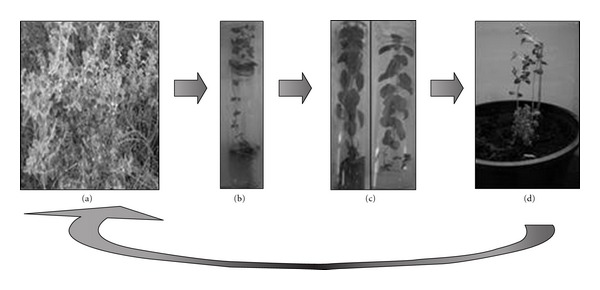
*In vitro* plant regeneration from single node of *C. odorum*. (a) General aspect of the wild plant in its habitat, (b) *in vitro* germinated plant after 10 weeks on MS medium, (c) *in vitro* grown plant after 2 months of culture on WP-2 medium, and (d) acclimatized plant, 1 month after potting into a mixture of garden soil and sand (2 : 1) subtracts.

Plantlets were acclimatized successfully when transferred to trays containing A mix substrate of plastic cups containing autoclaved garden soil and sand (2 : 1). Rooted plantlets were transferred into pots and the survival rate was the 85% after 1 month (Figure 1(d)). 

## 4. Conclusion

In conclusion, an efficient protocol for micropropagation of an important medicinal plant *C. odorum* was developed by testing various concentrations of growth regulators and nutrition conditions. The success of plant tissue culture as a mean of plant propagation is greatly influenced by nature of the culture medium used; they are grown *in vitro* on artificial media, which supplies the nutrients necessary for growth.

Different nutrient media hormone-free tested to find out the suitable starting nutrient medium supplemented with 3% (w/v) sucrose, for 8 weeks for *in vitro* nodal cuttings establishment, showed that an efficient survival percentage was obtained on plantlets grown on free-hormone WP medium. The free-hormone medium contains the proportion of inorganic nutrients to satisfy the nutritional as well as the physiological needs of this species *in vitro* culture. However, the optimum growth depended on the addition of PGRs. The positive response to these additions indicated a requirement of the explants for a morphogenetic induction as was observed when the explants grown on 1*⁄*2 B5-3, this medium appear to be adequate for new shoots formation.

 Our investigation revealed that no auxin supplementation was necessary for rooting differentiation (100%) in proliferation of regenerated shoots. 

The *in vitro*-grown plants could be transferred to ex vitro conditions, with a view to develope longer-term strategies for the transfer and reintroduction of micropropagated *C. odorum* plants into natural habitat [26, 27]. The results will make the conservation and propagation of the species much easier.

## Figures and Tables

**Table 1:** Effect of pregermination conditions on *in* *vitro* *C*. *odorum* culture, (A) control, (B) washing overnight in running tap water, (C) soaking at 1 mg L^−1^ GA_3_ during 12 h, (D) soaking at 1 mg L^−1^ of GA_3_ during 12 h, (E) soaking at 100 mg L^−1^ of GA_3_ during 12 h, (F) washing overnight in running tap water + soaking at 1 mg L^−1^ GA_3_ during 12 h, (G) washing overnight in running tap water + soaking at 10 mg L^−1^ GA_3_ during 12 h, and (H) washing overnight in running tap water + soaking at 100 mg L^−1^ GA_3_ during 12 h.

Pregermination condition	Percentage of germination
A	28.1 %
B	41.8 %
C	60.2 %
D	37.9 %
E	55.8 %
F	62.2 %
G	42.9 %
H	54.8 %

**Table 2:** Percentage of survival (%) of *C*. *odorum* explants on different culture media (1/2 MS, SH, WP, 1/2 B5) with the addition of different concentrations and combination of PGRs.

PGRs (mg L^−1^)	Culture media			
BAP/NAA	1/2 MS	SH	WP	1/2 B5
0.00	72.22	62.50	100.00	15.78
0.5/0.5	87.50	70.00	84.61	45.00
0.5/1.00	77.77	50.00	93.75	40.00
0.5/1.50	62.50	28.57	57.14	45.00
1.00/0.5	53.84	62.50	38.88	21.05
1.00/1.00	40.00	63.63	53.33	0.00
1.00/1.50	88.88	50.00	83.33	31.57
1.50/0.50	37.50	40.00	61.90	30.43
1.50/1.00	52.63	0.00	15.78	5.26
1.50/1.50	0.00	33.33	33.33	31.57

**Table 3:** Effect of nutrient media and PGRs (mg ^−1^) concentrations on *in* *vitro* *C*. *odorum*. Values followed by the same letter within a column are not significantly different at the *P* ≤ 0.05, according ANOVA analysis and by Duncan's test.

Culture media	BAP	NAA	Principal shoot length (cm)	Number of nodes (principal shoot)	Number of axillaries shoots
1/2 MS-0	0.00	0.00	6.06 ± 0.71^a^	9.46 ± 0.91^a^	1.00 ± 0.35^a^
1/2 MS-1	0.50	0.50	8.91 ± 0.68^a^	11.14 ± 0.88^a^	0.79 ± 0.34^a^
1/2 MS-2	0.50	1.00	9.81 ± 0.68^a^	11.93 ± 0.88^a^	0.79 ± 0.34^a^
1/2 MS-3	0.50	1.50	7.13 ± 0.81^a^	7.89 ± 1.09^a^	0.44 ± 0.43^a^
1/2 MS-4	1.00	0.50	3.91 ± 0.96^a^	5.14 ± 1.24^a^	1.00 ± 0.48^a^
1/2 MS-5	1.00	1.00	4.69 ± 0.90^a^	7.89 ± 1.09^a^	1.22 ± 0.43^a^
1/2 MS-6	1.00	1.50	12.14 ± 0.64^b^	16.38 ± 0.82^b^	1.75 ± 0.32^a^
1/2 MS-7	1.50	0.50	2.70 ± 1.04^a^	4.17 ± 1.34^a^	1.50 ± 0.52^a^
1/2 MS-8	1.50	1.00	5.22 ± 0.81^a^	7.50 ± 1.04^a^	3.60 ± 0.40^a^
1/2 MS-9	1.50	1.50	—	—	—

SH-0	0.00	0.00	7.46 ± 1.14^a^	6.60 ± 1.47^a^	1.80 ± 0.57^a^
SH-1	0.50	0.50	8.89 ± 0.96^a^	10.14 ± 1.24^b^	1.29 ± 0.48^a^
SH-2	0.50	1.00	8.60 ± 1.47^a^	10.00 ± 1.89^a^	1.67 ± 0.74^a^
SH-3	0.50	1.50	4.15 ± 1.80^a^	4.50 ± 1.32^a^	1.00 ± 0.90^a^
SH-4	1.00	0.50	3.56 ± 1.14^a^	4.40 ± 1.47^a^	0.80 ± 0.57^a^
SH-5	1.00	1.00	6.84 ± 0.96^a^	7.71 ± 1.24^a^	0.71 ± 0.48^a^
SH-6	1.00	1.50	5.85 ± 1.04^a^	8.33 ± 1.34^a^	1.17 ± 0.52^a^
SH-7	1.50	0.50	4.38 ± 1.27^a^	6.25 ± 1.64^a^	1.03 ± 0.48^a^
SH-8	1.50	1.00	—	—	—
SH-9	1.50	1.50	6.20 ± 1.47^a^	8.33 ± 1.89^a^	4.67 ± 0.74^a^

WP-0	0.00	0.00	7.74 ± 0.68^a^	7.71 ± 0.88^a^	0.93 ± 0.34^a^
WP-1	0.50	0.50	11.24 ± 0.77^a^	12.91 ± 0.99^a^	1.27 ± 0.39^a^
WP-2	0.50	1.00	12.67 ± 0.66^b^	13.07 ± 0.85^a^	1.53 ± 0.33^a^
WP-3	0.50	1.50	12.61 ± 0.74^b^	14.42 ± 0.95^a^	1.17 ± 0.37^a^
WP-4	1.00	0.50	9.39 ± 0.96^a^	9.71 ± 1.24^a^	0.29 ± 0.42^a^
WP-5	1.00	1.00	10.19 ± 0.90^a^	10.38 ± 1.16^a^	1.25 ± 0.45^a^
WP-6	1.00	1.50	9.14 ± 0.81^a^	10.50 ± 1.04^a^	0.80 ± 0.40^a^
WP-7	1.50	0.50	5.74 ± 0.71^a^	8.85 ± 0.91^a^	0.69 ± 0.35^a^
WP-8	1.50	1.00	2.57 ± 1.47^a^	6.33 ± 1.89^a^	0.33 ± 0.74^a^
WP-9	1.50	1.50	2.55 ± 1.27^a^	6.25 ± 1.64^a^	0.75 ± 0.64^a^

1/2 B5-0	0.00	0.00	2.37 ± 1.44^a^	4.33 ± 1.77^a^	0.67 ± 1.00^a^
1/2 B5-1	0.50	0.50	3.70 ± 0.83^a^	4.33 ± 1.02^a^	5.33 ± 0.58^a^
1/2 B5-2	0.50	1.00	6.21 ± 0.88^a^	6.63 ± 1.08^a^	6.63 ± 0.61^a^
1/2 B5-3	0.50	1.50	4.93 ± 0.83^a^	6.78 ± 1.02^a^	7.00 ± 0.58^b^
1/2 B5-4	1.00	0.50	4.72 ± 1.25^a^	7.00 ± 1.53^a^	6.75 ± 0.87^a^
1/2 B5-5	1.00	1.00	—	—	—
1/2 B5-6	1.00	1.50	6.12 ± 1.02^a^	6.83 ± 1.25^a^	3.00 ± 0.71^a^
1/2 B5-7	1.50	0.50	2.39 ± 0.94^a^	3.71 ± 1.16^a^	4.14 ± 0.65^a^
1/2 B5-8	1.50	1.00	1.90 ± 2.49^a^	5.00 ± 3.06^b^	—
1/2 B5-9	1.50	1.50	4.03 ± 1.02^a^	4.33 ± 1.25^a^	3.00 ± 0.71^a^

## Abbreviations

BAP: 6-Benzylaminopurine
B5: Gamborg medium
GA3: Gibberellic acid
IBA: Indole-3-butyric acid
NAA: 2-Naphthalene acetic acid
PGRs: Plant growth regulators
MS: Murashige and Skoog medium
SH: Schenk and Hildebrandt medium
WP: Woody plant medium (Lloyd and McCown) medium
1*⁄*2MS: Half-strength Murashige and Skoog macronutrients
1*⁄*2B5: Half-strength Gamborg macronutrients.
